# National, Regional, State, and Selected Local Area Vaccination Coverage Among Adolescents Aged 13–17 Years — United States, 2016

**DOI:** 10.15585/mmwr.mm6633a2

**Published:** 2017-08-25

**Authors:** Tanja Y. Walker, Laurie D. Elam-Evans, James A. Singleton, David Yankey, Lauri E. Markowitz, Benjamin Fredua, Charnetta L. Williams, Sarah A. Meyer, Shannon Stokley

**Affiliations:** ^1^Immunization Services Division, National Center for Immunization and Respiratory Diseases, CDC; ^2^Division of Viral Diseases, National Center for Immunization and Respiratory Diseases, CDC; ^3^Division of Bacterial Diseases, National Center for Immunization and Respiratory Diseases, CDC.

The Advisory Committee on Immunization Practices (ACIP) recommends that adolescents routinely receive tetanus, diphtheria, and acellular pertussis vaccine (Tdap), meningococcal conjugate vaccine (MenACWY), and human papillomavirus (HPV) vaccine (*1*) at age 11–12 years. ACIP also recommends catch-up vaccination with hepatitis B vaccine, measles, mumps, and rubella (MMR) vaccine, and varicella vaccine for adolescents who are not up to date with childhood vaccinations. ACIP recommends a booster dose of MenACWY at age 16 years (*1*). In December 2016, ACIP updated HPV vaccine recommendations to include a 2-dose schedule for immunocompetent adolescents initiating the vaccination series before their 15th birthday (*2*). To estimate adolescent vaccination coverage in the United States, CDC analyzed data from the 2016 National Immunization Survey–Teen (NIS-Teen) for 20,475 adolescents aged 13–17 years.* During 2015–2016, coverage increased for ≥1 dose of Tdap (from 86.4% to 88.0%) and for each HPV vaccine dose (from 56.1% to 60.4% for ≥1 dose). Among adolescents aged 17 years, coverage with ≥2 doses of MenACWY increased from 33.3% to 39.1%. In 2016, 43.4% of adolescents (49.5% of females; 37.5% of males) were up to date with the HPV vaccination series, applying the updated HPV vaccine recommendations retrospectively.^†^ Coverage with ≥1 HPV vaccine dose varied by metropolitan statistical area (MSA) status and was lowest (50.4%) among adolescents living in non-MSA areas and highest (65.9%) among those living in MSA central cities.^§^ Adolescent vaccination coverage continues to improve overall; however, substantial opportunities exist to further increase HPV-associated cancer prevention.

NIS-Teen is an annual survey that collects data on vaccines received by adolescents aged 13–17 years in the 50 states, the District of Columbia, selected local areas, and territories.^¶^ NIS-Teen is conducted among parents and guardians of eligible adolescents identified using a random-digit–dialed sample of landline and cellular telephone numbers.** Parents and guardians are interviewed for information on the sociodemographic characteristics of the adolescent and household, and contact information for the child’s vaccination providers. If more than one age-eligible adolescent lives in the household, one adolescent is randomly selected for participation. With parental/guardian consent, health care providers identified during the interview are mailed a questionnaire requesting the vaccination history from the adolescent’s medical record.^††^ This report presents vaccination coverage estimates for 20,475 adolescents (9,661 females and 10,814 males) aged 13–17 years with adequate provider data.^§§^ NIS-Teen methodology, including methods for weighting and synthesizing provider-reported vaccination histories, has been described (https://www.cdc.gov/vaccines/imz-managers/nis/downloads/NIS-PUF15-DUG.pdf). T-tests were used for statistical comparison of weighted data to account for the complex survey design. Weighted linear regression by survey year was used to estimate annual percentage point increases. Differences were considered statistically significant for p-values <0.05.

## National Vaccination Coverage

**FIGURE 1:**
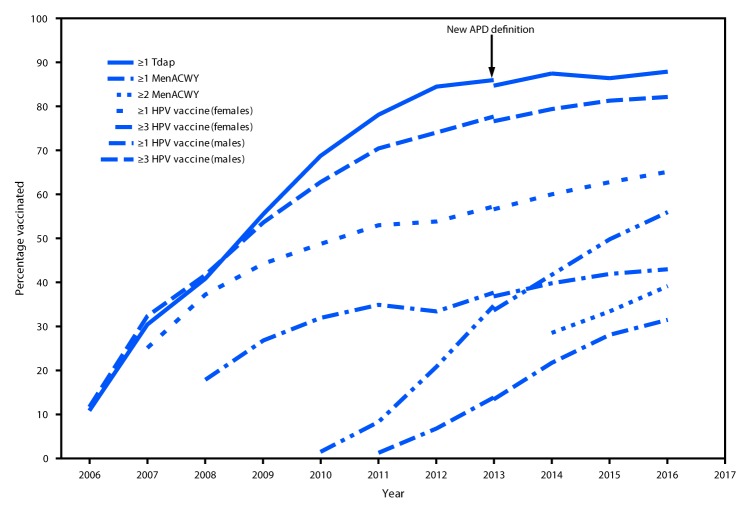
Estimated vaccination coverage with selected vaccines and doses* among adolescents aged 13–17 years, by survey year — National Immunization Survey–Teen (NIS-Teen), United States, 2006–2016^†^ **Abbreviations:** ACIP = Advisory Committee on Immunization Practices; APD = adequate provider data; HPV = human papillomavirus; MenACWY = quadrivalent meningococcal conjugate vaccine; Tdap = tetanus toxoid, reduced diphtheria toxoid, and acellular pertussis vaccine. * ≥1 dose Tdap at or after age 10 years; MenACWY: ≥2 doses MenACWY or meningococcal-unknown type vaccine, calculated only among adolescents aged 17 years at time of interview. Does not include adolescents who received their first and only dose of MenACWY at or after age 16 years; HPV vaccine, nine-valent (9vHPV), quadrivalent (4vHPV) or bivalent (2vHPV). ACIP recommends 9vHPV, 4vHPV or 2vHPV for females and 9vHPV or 4vHPV for males. The routine ACIP recommendation was made for females in 2006 and for males in 2011. ^†^ NIS-Teen implemented a revised APD definition in 2014, and retrospectively applied the revised APD definition to 2013 data. Estimates using different APD definitions might not be directly comparable.

In 2016, ≥1-dose HPV vaccination coverage among teens was 60.4% (65.1% for females; 56.0% for males), and 43.4% were up to date with the recommended HPV vaccination series (49.5% for females; 37.5% for males) (Table 1). During 2015–2016, HPV vaccination coverage increased for ≥1 dose by 4.3 percentage points overall (6.2 for males), for ≥2 doses by 3.8 percentage points (2.8 for females; 4.6 for males), and for ≥3 doses by 2.2 percentage points (3.4 for males) (Table 1) (Figure 1). Also during 2015–2016, coverage with ≥1 Tdap dose increased by 1.6 percentage points to 88.0%; among adolescents without a history of varicella disease, coverage with ≥2 varicella vaccine doses increased by 2.5 percentage points to 85.6%; and among persons aged 17 years, coverage with ≥2 MenACWY doses increased by 5.8 percentage points to 39.1% (Table 1) (Figure 1).

**TABLE 1:** Estimated vaccination coverage with selected vaccines and doses among adolescents aged 13–17* years, by age at interview — National Immunization Survey–Teen, United States, 2016

Vaccine	% (95% CI)^†^						
	Age (yrs)					Total	
	13	14	15	16	17	2016	2015
	(n = 4,209)	(n = 4,256)	(n = 4,113)	(n = 4,190)	(n = 3,707)	(N = 20,475)	(N = 21,875)
**Tdap^§^ ≥1 dose**	87.6 (85.4–89.6)	88.5 (86.3–90.4)	87.9 (85.5–89.9)	89.2 (87.5–90.7)	86.8 (84.4–88.9)	88.0 (87.1–88.9)^¶^	86.4 (85.4–87.3)
MenACWY**							
≥1 dose	81.7 (79.2–83.9)	83.3 (81.1–85.4)	80.4 (77.8–82.8)	82.3 (80.1–84.3)	83.5 (81.3–85.5)	82.2 (81.2–83.2)	81.3 (80.2–82.3)
≥2 doses^††^	—	—	—	—	39.1 (36.1–42.1)	39.1 (36.1–42.1)^¶^	33.3 (30.7–36.0)
**HPV^§§^ vaccine**							
**All adolescents**							
≥1 dose	53.5 (50.8–56.2)	59.2 (56.3–62.0)^¶^	62.0 (59.1–64.7)^¶^	61.9 (59.4–64.4)^¶^	65.4 (62.5–68.1)^¶^	60.4 (59.2–61.6)^¶^	56.1 (54.9–57.4)
≥2 doses	40.6 (37.9–43.4)	47.2 (44.2–50.2)^¶^	50.3 (47.4–53.3)^¶^	52.4 (49.8–55.0)^¶^	55.1 (52.1–58.1)^¶^	49.2 (47.9–50.4)^¶^	45.4 (44.2–46.7)
≥3 doses	27.0 (24.5–29.6)	34.9 (32.0–38.0)^¶^	37.6 (34.9–40.4)^¶^	42.9 (40.3–45.5)^¶^	43.1 (40.1–46.1)^¶^	37.1 (35.9–38.4)^¶^	34.9 (33.7–36.1)
HPV UTD***	33.7 (31.1–36.5)	42.5 (39.5–45.6)^¶^	45.4 (42.5–48.3)^¶^	47.6 (45.0–50.3)^¶^	47.3 (44.3–50.3)^¶^	43.4 (42.1–44.7)	NA
**Females**							
≥1 dose	54.7 (50.9–58.4)	62.7 (58.5–66.7)^¶¶^	68.4 (64.2–72.2)^¶¶^	66.8 (63.3–70.2)^¶¶^	72.7 (68.9–76.2)^¶¶^	65.1 (63.3–66.8)	62.8 (61.0–64.5)
≥2 doses	42.9 (39.1–46.8)	50.2 (45.7–54.6)^¶¶^	57.4 (52.8–61.8)^¶¶^	59.3 (55.7–62.9)^¶¶^	65.1 (61.0–69.0)^¶¶^	55.0 (53.1–56.8)^¶^	52.2 (50.3–54.0)
≥3 doses	28.8 (25.2–32.6)	38.4 (34.1–42.9)^¶¶^	43.7 (39.4–48.2)^¶¶^	50.0 (46.3–53.8)^¶¶^	54.2 (49.7–58.6)^¶¶^	43.0 (41.1–44.9)	41.9 (40.1–43.7)
HPV UTD	36.1 (32.4–40.0)	46.1 (41.6–50.5)^¶¶^	52.4 (47.8–56.9)^¶¶^	54.2 (50.5–57.9)^¶¶^	59.0 (54.6–63.3)^¶¶^	49.5 (47.6–51.4)	NA
**Males**							
≥1 dose	52.4 (48.5–56.3)	56.0 (52.0–59.9)	55.4 (51.7–59.0)	57.3 (53.7–60.8)	58.6 (54.6–62.6)^¶¶^	56.0 (54.3–57.7)^¶^	49.8 (48.0–51.6)
≥2 doses	38.4 (34.6–42.3)	44.5 (40.4–48.6)^¶¶^	43.1 (39.5–46.7)	45.9 (42.3–49.5)^¶¶^	45.9 (41.8–50.0)^¶¶^	43.6 (41.9–45.3)^¶^	39.0 (37.3–40.8)
≥3 doses	25.2 (21.9–28.8)	31.8 (27.9–36.0)^¶¶^	31.3 (28.1–34.7)^¶¶^	36.2 (32.8–39.8)^¶¶^	32.8 (29.3–36.6)^¶¶^	31.5 (30.0–33.2)^¶^	28.1 (26.6–29.7)
HPV UTD	31.4 (27.9–35.3)	39.3 (35.2–43.5)^¶¶^	38.2 (34.7–41.7)^¶¶^	41.4 (37.9–45.0)^¶¶^	36.6 (32.9–40.4)	37.5 (35.8–39.2)	NA
MMR vaccine ≥2 doses	90.7 (88.6–92.4)	91.9 (90.3–93.3)	91.4 (89.7–92.8)	91.1 (89.7–92.3)	89.4 (87.2–91.2)	90.9 (90.1–91.6)	90.7 (89.9–91.4)
Hepatitis B vaccine ≥3 doses	91.7 (89.7–93.3)	92.5 (91.0–93.8)	91.3 (89.5–92.8)	91.2 (89.8–92.5)	90.3 (88.2–92.0)	91.4 (90.7–92.1)	91.1 (90.2–91.9)
**Varicella**							
History of varicella^†††^	10.2 (8.8–11.8)	12.4 (10.8–14.2)	14.8 (13.0–16.9)^¶¶^	17.9 (16.0–20.1)^¶¶^	20.5 (18.1–23.2)^¶¶^	15.2 (14.3–16.1)^¶^	17.8 (16.8–18.9)
No history of varicella disease							
≥1 dose vaccine	95.0 (93.0–96.5)	96.2 (95.0–97.0)	94.8 (92.4–96.4)	94.9 (93.5–95.9)	94.0 (92.1–95.5)	95.0 (94.2–95.6)	94.9 (94.1–95.6)
≥2 doses vaccine	89.3 (87.0–91.2)	87.5 (85.2–89.6)	84.3 (81.5–86.8)^¶¶^	83.5 (81.4–85.5)^¶¶^	82.7 (80.2–84.9)^¶¶^	85.6 (84.5–86.6)^¶^	83.1 (82.0–84.2)
History of varicella or received ≥2 doses varicella vaccine	90.4 (88.3–92.1)	89.1 (87.0–90.9)	86.7 (84.2–88.8)^¶¶^	86.5 (84.7–88.1)^¶¶^	86.2 (84.2–88.0)^¶¶^	87.8 (86.9–88.6)^¶^	86.1 (85.2–87.0)

**Abbreviations:** CI = confidence interval; HPV = human papillomavirus; MenACWY = quadrivalent meningococcal conjugate vaccine; MMR = measles, mumps, and rubella; NA = not applicable – the update to the HPV recommendation occurred in December 2016; the new criteria was only applied retrospectively to the most current data year in which the recommendation was published; NIS-Teen = National Immunization Survey–Teen; Tdap = tetanus toxoid, reduced diphtheria toxoid, and acellular pertussis vaccine; UTD = up to date.

* Adolescents (N = 20,475) in the 2016 NIS-Teen were born during January 1998–February 2004.

^†^ Estimates with 95% CI half-widths >10 might not be reliable.

^§^ Includes percentages receiving Tdap vaccine at age ≥10 years.

^¶^ Statistically significant difference (p<0.05) compared with 2015 NIS-Teen estimates.

** Includes percentages receiving MenACWY or meningococcal-unknown type vaccine.

^††^ ACIP recommends a booster dose at age 16 years. Estimates are provided for ≥2 doses of MenACWY or meningococcal-unknown type vaccine. Calculated only among adolescents who were aged 17 years at time of interview. Does not include adolescents who received 1 dose of MenACWY vaccine at age ≥16 years.

^§§^ HPV vaccine, nine-valent (9vHPV), quadrivalent (4vHPV), or bivalent (2vHPV). For ≥1, ≥2, and ≥3 dose measures, percentages are reported among females and males combined (N = 20,475) and for females only (n = 9,661) and males only (n = 10,814).

^¶¶^ Statistically significant difference (p<0.05) in estimated vaccination coverage by age: reference group was adolescents aged 13 years.

***HPV UTD includes those who received ≥3 doses, and those who received 2 doses when the first HPV vaccine dose was initiated before age 15 years and the time between the first and second dose was at least 5 months minus 4 days.

^†††^ By parent/guardian report or provider records.

## Vaccination Coverage by Selected Characteristics

Tdap and MenACWY coverage was similar for each age group. For HPV vaccination (≥1-dose, ≥2-dose, and ≥3-dose coverage, and up-to-date status) coverage was higher overall and by sex, for persons aged 17 years (e.g., ≥1-dose coverage was 65.4% versus 53.5% at age 13 years), except for HPV up-to-date status among males, which was highest among males aged 16 years (Table 1). Among adolescents aged 13 years, HPV vaccination coverage was similar for females and males; among adolescents aged 17 years, HPV vaccination coverage was 14–23 percentage points higher among females than among males (Table 1).

Differences in vaccination coverage by race/ethnicity in 2016 were similar to patterns observed in previous years (Supplementary Table 1, https://stacks.cdc.gov/view/cdc/47510) (*3*). Coverage with Tdap, MenACWY, MMR vaccine, hepatitis B vaccine, and ≥2 doses of varicella vaccine did not differ by poverty status^¶¶^ (Table 2); however, HPV coverage, overall and by sex, was higher among adolescents living below the federal poverty level than among those living at or above the poverty level (e.g., overall, 12.9 percentage points and 8.4 percentage points higher for ≥1-dose coverage and up-to-date status, respectively). HPV coverage, overall and by sex, was 13–17 percentage points lower for adolescents living in non-MSA areas and 5–8 percentage points lower among those living in MSA non-central city areas than among those living in MSA central cities (Table 2). Coverage with ≥1 MenACWY dose and ≥2 varicella vaccine doses were 9.5 percentage points and 4.5 percentage points, respectively, lower among adolescents living in non-MSA areas than among those living in MSA central cities. Adolescents living in non-MSA areas were more likely to have all reported vaccination providers from public facilities (30.4%) than were those living in MSA non-central cities (10.3%) or MSA central cities (14.4%) (Supplementary Table 2, https://stacks.cdc.gov/view/cdc/47510).

**TABLE 2:** Estimated vaccination coverage with selected vaccines and doses among adolescents aged 13–17 years,* by poverty level^†^ and metropolitan statistical area (MSA)^§^ — National Immunization Survey–Teen, United States, 2016.

Vaccine	% (95% CI)^¶^							
	Poverty status			MSA				
	Below poverty level	At or above poverty level	Difference	Non-MSA	MSA non-central city	MSA central city	Difference between non-MSA and MSA central city	Difference between MSA non-central city and central city
	(n = 3,461)	(n = 16,290)	(n = 19,751)	(n = 4,248)	(n = 8,248)	(n = 7,979)	(n = 12,227)	(n = 16,227)
Tdap** ≥1 dose	86.7 (84.6 to 88.6)	88.4 (87.3 to 89.4)	-1.7 (-3.9 to 0.5)	87.7 (86.0 to 89.3)	87.8 (86.3 to 89.2)	88.4 (86.9 to 89.7)	-0.6 (-2.8 to 1.5)	-0.6 (-2.6 to 1.5)
**MenACWY^††^**								
≥1 dose	82.9 (80.5 to 85.0)	82.0 (80.8 to 83.1)	0.9 (-1.7 to 3.4)	74.1 (71.8 to 76.2)	83.3 (81.8 to 84.7)	83.5 (81.8 to 85.1)	-9.5 (-12.2 to -6.7) ^§§^	-0.2 (-2.5 to 2.0)
≥2 doses^¶¶^	36.4 (29.5 to 43.9)	39.1 (35.9 to 42.4)	-2.7 (-10.7 to 5.2)	31.6 (26.0 to 37.8)	43.0 (38.5 to 47.6)	37.1 (32.5 to 42.0)	-5.5 (-13.2 to 2.1)	5.9 (-0.7 to 12.5)
HPV*** vaccine coverage								
**All adolescents**								
≥1 dose	70.2 (67.4 to 72.8)	57.3 (55.9 to 58.7)	12.9 (9.8 to 15.9)^§§^	50.4 (47.8 to 53.0)	58.5 (56.6 to 60.3)	65.9 (64.0 to 67.9)	-15.6 (-18.8 to -12.3)^§§^	-7.5 (-10.1 to -4.8)^§§^
≥2 doses	55.9 (52.9 to 58.9)	47.1 (45.7 to 48.6)	8.8 (5.4 to 12.1)^§§^	38.5 (36.0 to 41.0)	48.0 (46.1 to 49.9)	54.0 (51.8 to 56.1)	-15.5 (-18.8 to -12.2)^§§^	-6.0 (-8.9 to -3.2)^§§^
≥3 doses	41.9 (38.9 to 44.9)	36.2 (34.8 to 37.6)	5.7 (2.3 to 9.0)^§§^	28.6 (26.4 to 31.0)	36.0 (34.2 to 37.8)	41.3 (39.1 to 43.4)	-12.6 (-15.8 to -9.5)^§§^	-5.3 (-8.1 to -2.5) ^§§^
HPV UTD^†††^	50.1 (47.0 to 53.1)	41.7 (40.3 to 43.1)	8.4 (5.0 to 11.7)^§§^	33.3 (30.9 to 35.8)	42.1 (40.2 to 43.9)	48.1 (46.0 to 50.3)	-14.8 (-18.1 to -11.5)^§§^	-6.1 (-8.9 to -3.2) ^§§^
**Females**								
≥1 dose	74.8 (71.0 to 78.2)	62.0 (60.0 to 63.9)	12.8 (8.7 to 16.8)^§§^	56.2 (52.5 to 59.9)	62.6 (60.0 to 65.2)	70.9 (68.1 to 73.5)	-14.6 (-19.2 to -10.0)^§§^	-8.2 (-12.0 to -4.4)^§§^
≥2 doses	63.8 (59.7 to 67.7)	52.8 (50.7 to 54.8)	11.0 (6.5 to 15.6)^§§^	45.3 (41.5 to 49.1)	53.2 (50.4 to 55.9)	60.3 (57.2 to 63.3)	-15.0 (-19.8 to -10.1)^§§^	-7.1 (-11.2 to -2.9)^§§^
≥3 doses	48.0 (43.7 to 52.3)	42.4 (40.3 to 44.5)	5.6 (0.8 to 10.4)^§§^	34.1 (30.7 to 37.7)	41.5 (38.8 to 44.2)	47.7 (44.5 to 50.9)	-13.5 (-18.3 to -8.7)^§§^	-6.2 (-10.4 to -2.0)^§§^
HPV UTD	58.1 (53.9 to 62.2)	47.9 (45.8 to 50.0)	10.2 (5.5 to 14.8)^§§^	39.4 (35.7 to 43.2)	47.4 (44.6 to 50.2)	55.5 (52.3 to 58.6)	-16.1 (-21.0 to -11.2)^§§^	-8.1 (-12.3 to -3.9)^§§^
**Males**								
≥1 dose	65.8 (61.7 to 69.6)	52.8 (50.9 to 54.7)	13.0 (8.6 to 17.4)^§§^	44.8 (41.2 to 48.4)	54.4 (51.8 to 56.9)	61.4 (58.5 to 64.1)	-16.5 (-21.1 to -12.0)^§§^	-7.0 (-10.8 to -3.2)^§§^
≥2 doses	48.4 (44.2 to 52.7)	41.8 (39.9 to 43.7)	6.6 (1.9 to 11.3)^§§^	32.0 (28.8 to 35.3)	42.8 (40.3 to 45.4)	48.2 (45.3 to 51.1)	-16.2 (-20.6 to -11.8)^§§^	-5.4 (-9.3 to -1.5)^§§^
≥3 doses	36.0 (32.0 to 40.3)	30.3 (28.6 to 32.1)	5.7 (1.2 to 10.3)^§§^	23.4 (20.6 to 26.4)	30.5 (28.3 to 32.9)	35.3 (32.6 to 38.2)	-12.0 (-16.0 to -7.9)^§§^	-4.8 (-8.4 to -1.2)^§§^
HPV UTD	42.5 (38.3 to 46.7)	35.8 (34.0 to 37.6)	6.7 (2.0 to 11.3)^§§^	27.6 (24.6 to 30.8)	36.8 (34.4 to 39.3)	41.3 (38.5 to 44.2)	-13.8 (-18.0 to -9.5)^§§^	-4.5 (-8.3 to -0.7)^§§^
≥2 MMR vaccine doses	90.5 (89.0 to 91.9)	91.1 (90.2 to 92.0)	-0.6 (-2.3 to 1.1)	90.3 (88.5 to 91.8)	90.9 (89.8 to 92.0)	91.1 (89.8 to 92.2)	-0.8 (-2.8 to 1.2)	-0.1 (-1.8 to 1.5)
≥3 Hepatitis B doses	90.2 (88.5 to 91.7)	91.9 (91.1 to 92.7)	-1.7 (-3.5 to 0.0)	91.1 (89.3 to 92.5)	92.0 (90.8 to 92.9)	90.9 (89.6 to 92.0)	0.2 (-1.8 to 2.2)	1.1 (-0.5 to 2.7)
**Varicella**								
History of varicella^§§§^	18.0 (15.8 to 20.5)	14.3 (13.3 to 15.3)	3.8 (1.3 to 6.3)^§§^	21.7 (19.4 to 24.1)	13.7 (12.5 to 15.0)	14.7 (13.3 to 16.4)	6.9 (4.1 to 9.7) ^§§^	-1.0 (-3.0 to 0.9)
Among adolescents with no history of varicella disease								
≥1 dose vaccine	95.2 (93.8 to 96.3)	95.1 (94.3 to 95.9)	0.0 (-1.4 to 1.5)	94.5 (92.7 to 95.8)	95.0 (93.8 to 96.0)	95.1 (93.9 to 96.0)	-0.6 (-2.5 to 1.3)	0.0 (-1.6 to 1.5)
≥2 doses vaccine	85.0 (82.5 to 87.2)	85.9 (84.8 to 87.0)	-1.0 (-3.5 to 1.6)	81.7 (79.2 to 83.9)	86.0 (84.4 to 87.5)	86.2 (84.5 to 87.8)	-4.5 (-7.4 to -1.7) ^§§^	-0.2 (-2.4 to 2.1)
History of varicella or received ≥2 doses varicella vaccine	87.7 (85.6 to 89.5)	88.0 (87.0 to 88.9)	-0.2 (-2.4 to 1.9)	85.6 (83.6 to 87.4)	87.9 (86.6 to 89.2)	88.2 (86.7 to 89.6)	-2.6 (-5.0 to -0.2) ^§§^	-0.3 (-2.2 to 1.7)

**Abbreviations:** CI = confidence interval; HPV = human papillomavirus; MenACWY = quadrivalent meningococcal conjugate vaccine; MMR = measles, mumps, and rubella; NIS-Teen = National Immunization Survey–Teen; Tdap = tetanus toxoid, reduced diphtheria toxoid, and acellular pertussis vaccine; UTD = up to date.

* Adolescents (N = 20,475) in the 2016 NIS-Teen were born during January 1998–February 2004.

^†^ Adolescents were classified as below poverty level if their total family income was less than the federal poverty level specified for the applicable family size and number of children aged <18 years. All others were classified as at or above the poverty level (https://www.census.gov/data/tables/time-series/demo/income-poverty/historical-poverty-thresholds.html). Poverty status was unknown for 724 adolescents.

^§^ MSA status was determined based on household-reported county of residence, and was grouped into three categories: MSA central city, MSA non-central city, and non-MSA. MSA and central city were as defined by the U.S. Census Bureau (https://www.census.gov/geo/reference/gtc/gtc_cbsa.html). Non-MSA areas include urban populations not located within an MSA as well as completely rural areas.

^¶^ Estimates with 95% CI half-widths >10 might not be reliable.

** Includes percentages receiving Tdap vaccine at age ≥10 years.

^††^ Includes percentages receiving MenACWY and meningococcal-unknown type vaccine.

^§§^ Statistically significant difference (p<0.05) in estimated vaccination coverage by poverty level or metropolitan statistical area; referent groups were adolescents living at or above poverty level and MSA central city, respectively.

^¶¶^ ≥2 doses of MenACWY or meningococcal-unknown type vaccine. Calculated only among adolescents aged 17 years at time of interview. Does not include adolescents who received 1 dose of MenACWY vaccine at age ≥16 years.

*** HPV vaccine, nine-valent (9vHPV), quadrivalent (4vHPV), or bivalent (2vHPV). For ≥1-, ≥2-, and ≥3-dose measures, percentages are reported among females and males combined (n = 20,475) and for females only (n = 9,661) and males only (n = 10,814).

^†††^ HPV UTD includes those with ≥3 doses, and those with 2 doses when the first HPV vaccine dose was initiated before age 15 years and time between the first and second dose was at least 5 months minus 4 days.

^§§§^ By parent/guardian report or provider records.

## State, Local, and Territorial Vaccination Coverage

**FIGURE 2:**
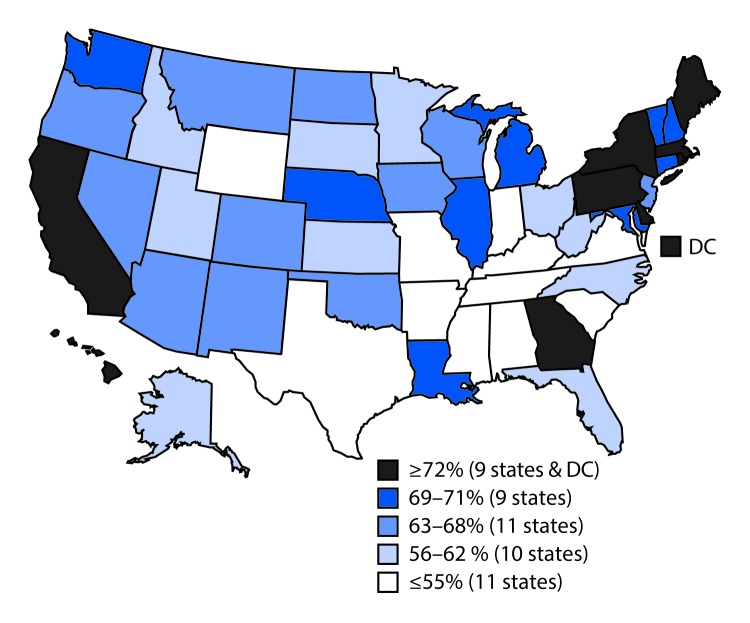
Estimated vaccination coverage* of ≥1 dose of human papillomavirus vaccine^†^ among female adolescents aged 13–17 years^§^^,^^¶^ —National Immunization Survey–Teen, United States, 2016 **Abbreviation:** DC = District of Columbia. * National coverage = 65%. ^†^ The Advisory Committee on Immunization Practices recommends nine-valent, quadrivalent, or bivalent HPV vaccine for females. ^§^ Sample size = 9,661. ^¶^ Includes female adolescents born during January 1998–February 2004.

**FIGURE 3:**
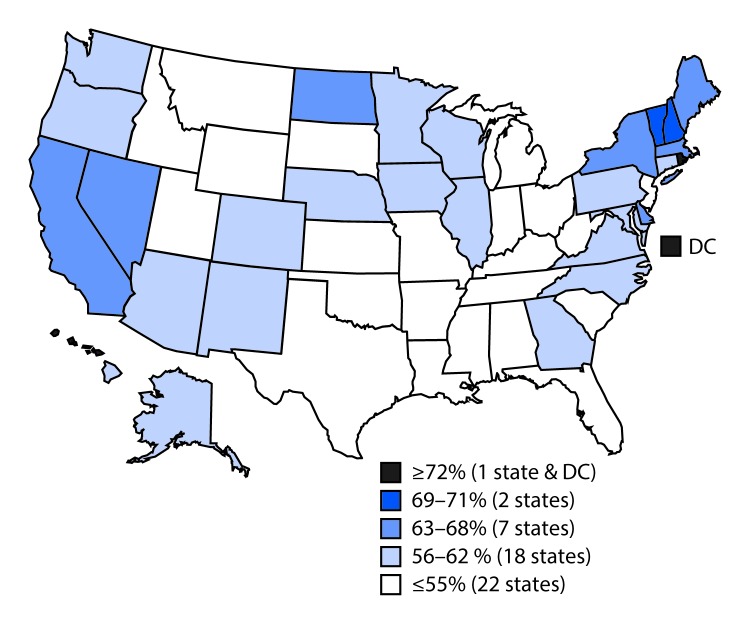
Estimated vaccination coverage* of ≥1 dose of human papillomavirus vaccine^†^ among male adolescents aged 13–17 years^§^^,^^¶^ — National Immunization Survey–Teen, United States, 2016 **Abbreviation:** DC = District of Columbia. *National coverage = 56%. ^†^ The Advisory Committee on Immunization Practices recommends nine-valent or quadrivalent HPV vaccine for males. ^§^ Sample size = 10,814. ^¶^ Includes male adolescents born during January 1998–February 2004.

Vaccination coverage varied by state (Table 3). For example, coverage with ≥1 Tdap dose ranged from 77.5% in South Carolina to 96.7% in Massachusetts, and ≥1-dose MenACWY coverage ranged from 54.2% in Wyoming to 96.4% in Rhode Island. Among females, ≥1-dose HPV vaccination coverage ranged from 47.8% in Mississippi to 90.1% in Rhode Island (Table 3) (Figure 2); among males, ≥1-dose HPV coverage ranged from 36.9% in Indiana and Wyoming to 87.8% in Rhode Island (Table 3) (Figure 3). HPV up-to-date estimates among females ranged from 30.8% in South Carolina to 73.0% in Rhode Island, and among males, from 19.9% in Wyoming to 68.7% in Rhode Island. During 2013–2016, ≥1-dose HPV vaccination coverage increased an average of 5.0 percentage points per year nationally; among states, local areas, and territories, the greatest statistically significant average annual increases were in New York City (7.7 percentage points), Nevada (7.6), Maryland (7.4), Guam (7.3), New York (7.2), and Alaska (7.1) (Supplementary Table 3, https://stacks.cdc.gov/view/cdc/47510).

**TABLE 3:** Estimated vaccination coverage with selected vaccines and doses* among adolescents aged 13–17 years,^†^ by HHS region, state, selected local area, or territory — National Immunization Survey–Teen, United States, 2016

Region, state, local area	% (95% CI)^§^							
	All adolescents (N = 20,475)				Females (n = 9,661)		Males (n = 10,814)	
	≥1 Tdap^¶^	≥1 MenACWY**	≥1 HPV^††^	HPV UTD^§§^	≥1 HPV^††^	HPV UTD^§§^	≥1 HPV^††^	HPV UTD^§§^
U.S. overall	88.0 (87.1–88.9) ^¶¶^	82.2 (81.2–83.2)	60.4 (59.2–61.6) ^¶¶^	43.4 (42.1–44.7)	65.1 (63.3–66.8)	49.5 (47.6–51.4)	56.0 (54.3–57.7) ^¶¶^	37.5 (35.8–39.2)
**Region I**	94.8 (93.4–96.0) ^¶¶^	90.8 (88.7–92.6)	69.9 (66.7–72.9)	55.0 (51.7–58.3)	74.9 (70.7–78.7)	61.0 (56.2–65.5)	65.1 (60.5–69.5)	49.3 (44.7–54.0)
Connecticut	93.9 (89.6–96.5)	93.9 (89.9–96.4)	62.2 (55.8–68.2)	49.0 (42.7–55.3)	68.9 (60.3–76.4)	56.9 (48.1–65.3)	55.8 (46.6–64.6)	41.5 (32.8–50.7)
Maine	87.5 (83.1–90.9)	83.5 (78.1–87.8)	70.0 (63.9–75.4)	56.0 (49.8–62.1)	73.1 (64.4–80.3)	64.3 (55.5–72.3)	67.1 (58.4–74.8)	48.2 (39.6–56.9)
Massachusetts	96.7 (94.4–98.0) ^¶¶^	90.4 (86.2–93.5)	71.4 (65.7–76.5)	56.6 (50.6–62.5)	77.6 (69.7–83.9)	62.0 (53.3–70.1)	65.5 (57.1–72.9)	51.4 (43.1–59.6)
New Hampshire	95.3 (91.5–97.5)	88.0 (83.1–91.6)	69.9 (63.7–75.5)	51.2 (44.6–57.8)	70.6 (61.9–78.1)	56.5 (47.3–65.2)	69.3 (60.1–77.1)	46.3 (36.9–55.9)
Rhode Island	95.4 (92.5–97.2)	96.4 (93.2–98.1)	88.9 (84.7–92.1)	70.8 (64.4–76.4)	90.1 (83.4–94.2)	73.0 (63.5–80.8)	87.8 (81.7–92.1)	68.7 (59.8–76.4)
Vermont	93.8 (90.4–96.1)	86.4 (82.0–89.9)	70.3 (64.6–75.5)	55.7 (49.9–61.3)	71.2 (62.4–78.6)	58.4 (49.7–66.7)	69.5 (61.8–76.3)	53.1 (45.2–60.7)
**Region II**	90.8 (88.4–92.7)	90.0 (87.6–92.0)	67.2 (63.8–70.5) ^¶¶^	51.4 (47.7–55.1)	72.0 (67.2–76.4) ^¶¶^	57.6 (52.3–62.8)	62.7 (57.6–67.4)	45.5 (40.5–50.7)
New Jersey	89.9 (85.5–93.1)	91.7 (87.9–94.4)	58.5 (52.4–64.3)	42.8 (37.0–48.8)	66.0 (57.8–73.4)	50.1 (41.8–58.5)	51.2 (42.7–59.7)	35.8 (28.2–44.1)
New York	91.1 (88.2–93.4)	89.2 (86.0–91.8)	71.5 (67.3–75.4) ^¶¶^	55.7 (51.0–60.2)	75.0 (69.0–80.1) ^¶¶^	61.3 (54.6–67.6)	68.2 (62.1–73.8)	50.3 (43.9–56.7)
NY–City of New York	88.9 (84.1–92.3)	89.6 (84.4–93.2)	76.8 (70.8–81.9)	61.7 (54.9–68.1)	81.9 (73.8–87.9) ^¶¶^	69.9 (60.5–77.8)	71.9 (62.9–79.5)	53.9 (44.2–63.2)
NY–Rest of state	92.6 (88.6–95.3)	89.0 (84.5–92.3)	68.1 (62.3–73.5) ^¶¶^	51.8 (45.6–58.0)	70.5 (62.0–77.8)	55.8 (46.7–64.5)	65.9 (57.6–73.3) ^¶¶^	48.0 (39.7–56.4)
**Region III**	88.9 (86.6–90.8)	84.7 (81.9–87.1)	61.2 (57.9–64.4)	46.9 (43.6–50.2)	65.0 (60.2–69.5)	51.9 (47.1–56.6)	57.6 (53.0–62.0)	42.1 (37.7–46.6)
Delaware	87.5 (83.0–91.0)	87.3 (82.4–91.0)	70.7 (64.9–75.8)	56.9 (50.7–62.8)	78.3 (70.5–84.5)	66.8 (58.4–74.3)	63.3 (54.7–71.1)	47.3 (38.8–55.9)
District of Columbia	86.5 (81.5–90.3)	86.9 (81.3–91.0)	79.2 (73.5–84.0)	62.0 (55.3–68.2)	80.7 (72.2–87.0)	65.1 (55.4–73.7)	77.7 (69.5–84.3)	58.8 (49.4–67.6)
Maryland	85.0 (79.7–89.2)	84.8 (79.0–89.3)	64.5 (58.1–70.5)	48.1 (41.6–54.6)	69.0 (59.9–76.8)	51.8 (42.6–60.9)	60.2 (51.0–68.7)	44.5 (35.6–53.7)
Pennsylvania	92.0 (88.9–94.2)	92.7 (89.6–94.9)	64.4 (59.3–69.2)	51.0 (45.9–56.1)	72.0 (65.1–78.1)	58.0 (50.6–65.1)	57.2 (49.9–64.1)	44.4 (37.5–51.5)
PA–Philadelphia	89.8 (85.4–93.0)	91.2 (87.1–94.1)	80.7 (75.4–85.0)	68.4 (62.5–73.8)	88.2 (81.2–92.8)	76.2 (67.8–83.0)	73.7 (65.7–80.3)	61.1 (52.8–68.9)
PA–Rest of state	92.3 (88.7–94.7)	92.9 (89.3–95.3)	62.3 (56.6–67.6)	48.7 (43.0–54.5)	69.9 (62.1–76.7)	55.6 (47.4–63.6)	54.9 (46.8–62.8)	42.1 (34.5–50.2)
Virginia	87.1 (81.0–91.5)	71.5 (63.9–78.1)	53.6 (46.0–61.0)	39.2 (32.1–46.8)	50.7 (39.6–61.6)	41.1 (30.8–52.3)	56.4 (46.2–66.0) ^¶¶^	37.4 (28.0–47.9)
West Virginia	89.7 (85.3–92.9)	89.0 (84.5–92.3)	54.2 (47.5–60.8)	41.2 (34.7–47.9)	58.5 (48.8–67.6)	49.7 (40.2–59.3)	50.0 (40.9–59.2)	33.0 (24.9–42.2)
**Region IV**	88.9 (87.1–90.5)	77.7 (75.4–79.9)	55.8 (53.1–58.5) ^¶¶^	38.7 (36.1–41.4)	59.6 (55.7–63.3)	44.8 (40.9–48.7)	52.3 (48.5–56.0) ^¶¶^	32.9 (29.3–36.6)
Alabama	91.7 (87.8–94.4)	72.4 (66.4–77.7)	51.7 (45.3–58.0)	35.4 (29.5–41.7)	54.2 (45.1–63.1)	46.5 (37.4–55.7)	49.2 (40.5–58.1)	24.7 (17.9–32.9)
Florida	89.7 (84.5–93.3)	76.3 (70.2–81.5)	55.9 (49.2–62.5)	40.4 (34.0–47.1)	58.4 (48.6–67.6)	46.4 (36.9–56.2)	53.5 (44.4–62.5)	34.5 (26.3–43.8)
Georgia	92.8 (88.3–95.6)	91.4 (87.1–94.4)	67.3 (60.9–73.2) ^¶¶^	45.6 (39.2–52.2)	77.0 (68.9–83.5) ^¶¶^	55.4 (46.2–64.2)	58.0 (48.5–67.0)	36.2 (27.8–45.6)
Kentucky	89.0 (84.6–92.2)	85.9 (81.2–89.6) ^¶¶^	48.0 (41.7–54.4)	34.0 (28.0–40.5)	54.8 (45.6–63.7)	39.7 (30.9–49.3)	41.6 (33.2–50.5)	28.5 (21.0–37.3)
Mississippi	82.0 (76.5–86.5)	57.4 (51.0–63.5)	45.6 (39.4–52.0)	29.1 (23.6–35.2)	47.8 (38.7–57.0)	33.9 (25.6–43.3)	43.6 (35.1–52.4)	24.5 (17.8–32.7)
North Carolina	89.1 (84.5–92.5)	75.7 (69.7–80.9)	57.5 (51.0–63.8)	41.2 (35.0–47.7)	57.9 (48.8–66.5)	46.9 (38.1–56.0)	57.1 (47.8–66.0)	35.7 (27.3–45.1)
South Carolina	77.5 (70.7–83.1)	68.9 (61.9–75.2)	44.2 (37.4–51.3)	29.1 (23.3–35.6)	50.5 (40.2–60.7)	30.8 (22.5–40.6)	38.2 (29.7–47.5)	27.4 (20.0–36.4)
Tennessee	89.3 (84.1–92.9) ^¶¶^	76.3 (70.0–81.7)	55.3 (48.5–61.9)	36.0 (29.7–42.8)	55.3 (45.4–64.7)	36.9 (28.1–46.6)	55.3 (46.1–64.3) ^¶¶^	35.2 (26.5–44.9)
**Region V**	91.2 (89.5–92.6) ^¶¶^	85.9 (84.0–87.7)	58.4 (55.8–61.0) ^¶¶^	43.4 (40.8–46.1)	63.4 (59.7–67.0)	49.2 (45.4–53.0)	53.7 (49.9–57.4) ^¶¶^	38.0 (34.4–41.7)
Illinois	91.0 (87.9–93.3)	83.9 (79.9–87.3)	63.5 (58.6–68.1) ^¶¶^	47.8 (42.9–52.7)	68.5 (61.7–74.5)	52.6 (45.6–59.5)	58.7 (51.8–65.3) ^¶¶^	43.2 (36.6–50.1)
IL–City of Chicago	84.2 (75.2–90.3)	91.1 (84.6–95.0) ^¶¶^	73.1 (63.2–81.2)	55.7 (45.6–65.3)	79.7 (66.0–88.8)	65.3 (51.5–76.9)	66.8 (52.0–79.0)	46.4 (32.8–60.6)
IL–Rest of state	92.5 (89.2–94.8)	82.3 (77.6–86.3)	61.4 (55.8–66.6) ^¶¶^	46.1 (40.6–51.7)	66.0 (58.2–73.0)	49.8 (41.8–57.7)	56.9 (49.2–64.4) ^¶¶^	42.6 (35.2–50.3)
Indiana	89.5 (84.6–92.9)	88.0 (82.7–91.8)	45.2 (38.9–51.7)	33.9 (28.0–40.2)	53.9 (44.5–63.1)	43.5 (34.3–53.0)	36.9 (29.0–45.5)	24.7 (18.1–32.8)
Michigan	93.6 (89.4–96.2) ^¶¶^	95.0 (91.8–97.0)	61.3 (54.2–67.9)	44.8 (37.9–51.9)	70.5 (60.9–78.7)	55.4 (45.5–65.0)	52.5 (42.6–62.2)	34.6 (26.1–44.3)
Minnesota	89.7 (85.0–93.1)	85.2 (80.1–89.1)	59.1 (53.0–65.0)	44.1 (38.1–50.3)	58.1 (49.0–66.7)	46.4 (37.7–55.3)	60.1 (51.6–68.0)	42.0 (33.9–50.6)
Ohio	90.8 (85.6–94.3)	79.6 (73.4–84.7)	56.2 (49.5–62.8)	41.8 (35.3–48.6)	57.6 (48.1–66.5)	42.5 (33.7–51.9)	55.0 (45.2–64.3)	41.1 (31.9–51.0)
Wisconsin	91.6 (87.2–94.5)	85.6 (80.7–89.4)	61.9 (55.5–67.9)	45.5 (39.2–52.0)	68.1 (58.6–76.2)	53.6 (44.1–63.0)	56.0 (47.3–64.4)	37.8 (29.7–46.5)
**Region VI**	86.7 (84.6–88.5)	84.9 (82.8–86.8)	52.0 (49.3–54.8)	35.0 (32.5–37.7)	57.3 (53.2–61.2)	41.3 (37.4–45.4)	47.0 (43.3–50.8)	28.9 (25.8–32.3)
Arkansas	91.0 (87.1–93.8)	89.1 (84.9–92.2) ^¶¶^	54.4 (48.1–60.5)	34.5 (28.9–40.7)	53.3 (43.8–62.6)	35.5 (27.1–45.0)	55.4 (47.3–63.3)	33.6 (26.3–41.7)
Louisiana	93.7 (89.8–96.2)	90.9 (86.9–93.8)	60.5 (54.4–66.3)	41.8 (35.8–48.1)	69.9 (61.1–77.4)	50.8 (41.7–59.8)	51.5 (43.1–59.9)	33.2 (25.7–41.7)
New Mexico	84.3 (79.2–88.4)	77.8 (72.5–82.4)	60.5 (54.4–66.3)	42.9 (37.0–49.0)	63.1 (54.4–71.1)	49.0 (40.4–57.7)	57.9 (49.3–66.1)	37.0 (29.3–45.3)
Oklahoma	89.6 (84.2–93.3)	73.6 (66.6–79.6)	56.9 (49.5–63.9)	39.2 (32.4–46.4)	63.8 (53.0–73.4)	43.6 (33.6–54.1)	50.3 (40.3–60.2)	35.0 (26.1–45.2)
Texas	85.0 (82.1–87.5)	85.5 (82.6–88.0) ***	49.3 (45.6–53.0)	32.9 (29.6–36.5)	54.5 (49.0–59.8)	39.7 (34.4–45.1)	44.3 (39.4–49.4)	26.5 (22.5–30.9)
TX–Bexar County	85.4 (80.1–89.5)	87.2 (81.8–91.2)	53.4 (46.7–59.9)	39.2 (33.0–45.8)	58.3 (48.6–67.5)	45.2 (35.8–55.0)	48.5 (39.7–57.4)	33.3 (25.6–42.1)
TX–City of Houston	86.2 (77.9–91.7)	82.9 (73.8–89.3)	62.6 (53.6–70.9)	46.4 (37.6–55.3)	59.4 (45.7–71.8)	44.2 (31.8–57.3)	65.9 (54.2–75.9)	48.6 (36.8–60.5)
TX–Dallas County	83.0 (76.7–87.9)	87.7 (82.0–91.8)	45.7 (38.5–53.1)	23.9 (18.8–30.0)	48.8 (37.9–59.8)	24.3 (16.8–33.6)	42.7 (33.4–52.5)	23.6 (16.9–32.0)
TX–El Paso County	83.4 (76.6–88.6)	91.6 (86.5–94.8) ^¶¶^	79.8 (73.6–84.9) ^¶¶^	66.0 (59.0–72.4)	78.4 (68.4–85.9)	69.0 (58.9–77.6)	81.1 (73.0–87.2) ^¶¶^	63.2 (53.2–72.1)
TX–Rest of state	85.2 (81.4–88.3)	85.0 (81.2–88.2) ***	46.8 (42.0–51.6)	30.7 (26.4–35.4)	53.3 (46.2–60.2)	39.2 (32.5–46.4)	40.6 (34.2–47.2)	22.5 (17.7–28.3)
**Region VII**	86.2 (83.6–88.4)	70.8 (67.4–74.0)	55.3 (51.8–58.8)	39.3 (36.0–42.8)	60.7 (55.7–65.5)	43.7 (38.8–48.7)	50.2 (45.3–55.1)	35.2 (30.6–40.0)
Iowa	89.2 (85.0–92.2)	74.9 (69.4–79.7)	60.7 (54.8–66.3)	45.5 (39.7–51.5)	64.4 (55.9–72.2)	47.4 (39.0–55.9)	57.2 (48.9–65.1)	43.8 (35.8–52.0)
Kansas	87.3 (82.1–91.2)	69.7 (63.5–75.3)	51.8 (45.2–58.3)	35.6 (29.6–42.1)	62.4 (53.1–70.9)	45.6 (36.6–54.9)	41.7 (32.9–51.0)	26.0 (18.7–35.0)
Missouri	83.9 (78.7–88.0)	66.2 (59.6–72.2)	51.6 (45.0–58.1)	35.8 (29.8–42.4)	55.0 (45.7–64.0)	38.5 (29.8–48.0)	48.3 (39.1–57.5)	33.3 (25.2–42.5)
Nebraska	86.8 (81.5–90.8)	80.2 (74.6–84.8)	63.7 (57.2–69.8)	45.9 (39.4–52.5)	69.4 (59.8–77.6)	50.6 (41.1–60.0)	58.3 (49.3–66.8)	41.3 (32.6–50.6)
**Region VIII**	85.9 (83.1–88.3)	75.4 (72.1–78.4)	57.4 (53.6–61.0)	40.6 (36.9–44.4)	64.1 (58.6–69.3)	48.1 (42.6–53.7)	50.9 (45.7–56.1)	33.5 (28.6–38.7)
Colorado	87.5 (82.1–91.4) ***	77.5 (71.3–82.7) ***	63.5 (56.5–69.9)	48.0 (41.0–55.0)	68.3 (57.9–77.1)	52.1 (41.9–62.2)	58.8 (49.2–67.8)	44.0 (34.7–53.7)
Montana	85.7 (80.4–89.7)	67.6 (61.3–73.3)	55.3 (48.9–61.5)	39.9 (33.8–46.4)	68.2 (59.6–75.7) ^¶¶^	52.5 (43.6–61.2)	43.0 (34.5–51.9)	27.9 (20.8–36.4)
North Dakota	92.0 (87.5–94.9)	92.0 (87.5–94.9)	67.6 (60.9–73.5)	52.7 (46.0–59.3)	68.3 (58.2–76.9)	60.2 (50.1–69.5)	66.9 (57.9–74.7)	45.5 (36.6–54.6)
South Dakota	79.4 (73.1–84.5)	65.7 (59.1–71.7) ^¶¶^	55.9 (49.2–62.3) ^¶¶^	38.6 (32.4–45.2)	61.7 (51.8–70.8)	47.3 (37.8–57.1)	50.4 (41.5–59.3)	30.5 (23.0–39.2)
Utah	83.9 (78.5–88.2)	76.6 (70.5–81.7)	49.7 (43.1–56.2)	30.5 (24.9–36.9)	58.8 (49.2–67.8)	41.3 (32.4–50.7)	40.9 (32.4–50.1)	20.3 (13.9–28.7)
Wyoming	86.7 (81.7–90.5)	54.2 (48.1–60.1)	43.4 (37.5–49.5)	26.7 (21.7–32.3)	50.4 (41.5–59.3)	33.9 (26.1–42.7)	36.9 (29.2–45.3)	19.9 (14.1–27.3)
**Region IX**	82.7 (77.9–86.6)	80.3 (75.5–84.4)	70.6 (65.6–75.2) ^¶¶^	48.0 (42.5–53.5)	75.3 (68.0–81.4)	55.8 (47.5–63.8)	66.1 (59.1–72.5)	40.5 (33.6–47.7)
Arizona	84.3 (78.7–88.6)	85.2 (79.7–89.3)	63.1 (56.6–69.2)	44.1 (37.5–51.0)	65.4 (56.1–73.6)	46.6 (37.2–56.2)	60.9 (51.6–69.5)	41.7 (32.7–51.3)
California	82.1 (75.9–86.9)	79.7 (73.5–84.8)	72.6 (66.1–78.2) ^¶¶^	49.1 (42.2–56.0)	78.0 (68.5–85.3)	58.3 (47.6–68.2)	67.3 (58.4–75.2)	40.3 (31.8–49.4)
Hawaii	82.2 (76.5–86.7)	75.8 (69.7–81.1)	64.8 (58.3–70.8)	54.0 (47.5–60.3)	71.7 (62.3–79.5)	61.5 (52.0–70.2)	58.3 (49.2–66.8)	46.9 (38.2–55.7)
Nevada	87.1 (81.8–91.0)	78.7 (72.8–83.7)	64.9 (58.5–70.8)	39.9 (33.7–46.5)	64.6 (55.6–72.7)	43.0 (34.2–52.3)	65.1 (55.9–73.3) ^¶¶^	37.0 (28.4–46.5)
**Region X**	85.5 (82.3–88.1)	75.0 (71.3–78.4)	62.6 (58.8–66.3) ^¶¶^	46.7 (42.8–50.7)	66.5 (61.1–71.5)	51.7 (46.0–57.4)	58.9 (53.5–64.1) ^¶¶^	41.9 (36.6–47.4)
Alaska	79.4 (74.1–83.9) ^¶¶^	67.0 (61.1–72.4) ^¶¶^	61.1 (55.0–66.9) ^¶¶^	43.3 (37.2–49.6)	61.9 (53.0–70.0)	47.8 (39.0–56.8)	60.3 (51.7–68.4) ^¶¶^	39.1 (30.9–48.0)
Idaho	87.5 (83.0–90.9)	86.5 (81.4–90.4)	57.2 (50.9–63.3)	36.5 (30.8–42.6)	59.8 (50.7–68.4)	43.4 (34.9–52.3)	54.7 (46.0–63.1)	30.0 (22.7–38.4)
Oregon	83.2 (77.0–88.0)	70.5 (64.2–76.2)	61.7 (55.2–67.9)	47.5 (41.0–54.0)	62.6 (52.7–71.6)	50.3 (40.7–59.9)	60.9 (52.1–69.0)	44.7 (36.1–53.7)
Washington	86.8 (81.5–90.7)	75.1 (68.9–80.5)	64.8 (58.5–70.6) ^¶¶^	49.5 (43.0–55.9)	70.9 (62.4–78.3)	55.2 (45.8–64.2)	58.9 (49.9–67.3)	44.0 (35.4–52.9)
**Range^†††^**	(77.5 – 96.7)	(54.2 – 96.4)	(43.4 – 88.9)	(26.7 – 70.8)	(47.8 – 90.1)	(30.8 – 73.0)	(36.9 – 87.8)	(19.9 – 68.7)
**Territory**								
Guam	77.5 (72.9–81.6)	77.1 (72.6–81.1)	67.4 (62.4–72.1)	44.2 (39.0–49.5)	76.9 (70.0–82.5)	55.8 (48.0–63.4)	58.5 (51.4–65.3)	33.2 (27.0–40.1)
Puerto Rico	91.2 (87.5–93.9) ^¶¶^	89.2 (85.0–92.3)	75.8 (70.2–80.6)	52.8 (46.4–59.0)	80.8 (72.7–86.9)	61.9 (52.8–70.3)	71.1 (62.9–78.1)	44.1 (35.7–53.0)
U.S. Virgin Islands	78.9 (73.7–83.2)	61.3 (55.5–66.7)	41.9 (36.2–47.7)	22.6 (17.9–28.1)	43.8 (35.6–52.4)	26.6 (19.5–35.0)	40.1 (32.5–48.1)	19.0 (13.2–26.4)

**Abbreviations:** CI = confidence interval; HHS = U.S. Department of Health and Human Services; HPV = human papillomavirus; MenACWY = quadrivalent meningococcal conjugate vaccine; NIS-Teen = National Immunization Survey–Teen; Tdap = tetanus toxoid, reduced diphtheria toxoid, and acellular pertussis vaccine; UTD = up to date.

* Estimates for additional measures, including MMR, hepatitis B, and varicella vaccines are available at https://www.cdc.gov/vaccines/vaxview/teenvaxview.

^†^ Adolescents (N = 20,475) in the 2016 NIS-Teen were born during January 1998—February 2004.

^§^ Estimates with 95% CI half-widths >10 might not be reliable.

^¶^ ≥1 dose Tdap vaccine at age ≥10 years.

** ≥1 dose of MenACWY or meningococcal-unknown type vaccine.

^††^ HPV vaccine, nine-valent (9vHPV), quadrivalent (4vHPV), or bivalent (2vHPV). For ≥1-, ≥2-, and ≥3-dose measures, percentages are reported among females and males combined (n = 20,475) and for females only (n = 9,661) and males only (n = 10,814).

^§§^ HPV UTD includes those with ≥3 doses, and those with 2 doses when the first HPV vaccine dose was initiated before age 15 years and time between the first and second dose was at least 5 months minus 4 days.

^¶¶^ Statistically significant (p<0.05) percentage point increase from 2015.

*** Statistically significant (p<0.05) percentage point decrease from 2015.

^†††^ Range excludes selected local areas and territories.

## Discussion

In 2016, adolescent vaccination coverage in the United States was sustained and continued to improve in several areas: compared with 2015, coverage with Tdap, ≥2 doses of varicella vaccine, ≥2 doses of MenACWY, and each dose of HPV vaccine increased. Since HPV vaccine was introduced for females in 2006 and for males in 2011, coverage has increased gradually among females and more rapidly among males. During 2015–2016, increases in coverage with each HPV dose, ranging from 3.4 to 6.2 percentage points occurred among males, whereas only a 2.8 percentage point increase in ≥2-dose HPV coverage occurred among females. Coverage with ≥1-dose HPV vaccine among males continues to approach that among females, particularly for adolescents aged 13 years, suggesting that HPV vaccination of both female and male adolescents has been integrated into vaccination practices. Although HPV vaccination initiation (receipt of ≥1 HPV vaccine dose) continues to increase, coverage remains 22–28 percentage points lower than those for Tdap and ≥1-dose MenACWY. These gaps indicate substantial opportunity for improving HPV vaccination practices.

Disparities in adolescent vaccination coverage were found by MSA status: HPV vaccination initiation among adolescents living outside MSA central cities was 16 percentage points lower than among those living in MSA central cities. Although adolescents living in non-MSA areas had substantially lower HPV and MenACWY vaccination coverage compared with those living in MSA central cities, Tdap coverage in these groups was similar. Reasons for these disparities are not well understood. Potential contributing factors might include differences in parental acceptance of certain vaccines and provider participation in, and adolescents’ eligibility for, the Vaccines for Children program.*** The disproportionately lower number of pediatric primary care providers found in non-MSA areas than in MSA central city areas (*4*,*5*) might partially explain this difference in vaccination coverage, because nonpediatric providers might be less familiar with adolescent vaccination recommendations. Because Tdap coverage is substantially higher than ≥1-dose HPV coverage, even in non-MSA areas, lack of access to any vaccination services is unlikely the underlying cause of lower HPV vaccine initiation. A better understanding of reasons for variations in HPV vaccine initiation by MSA status is needed to identify appropriate, targeted strategies to improve HPV vaccination coverage. CDC has published a series of reports in an effort to better understand health disparities between rural and urban areas (https://www.cdc.gov/ruralhealth/caseforruralhealth.html).

Variation in adolescent vaccination coverage among state and local areas might reflect differences in adolescent health care delivery, the prevalence of factors associated with lower vaccination coverage, and immunization program emphasis on, and effectiveness of, adolescent vaccination activities. Immunization programs in several state and local jurisdictions (e.g., Alaska, Maryland, Nevada, New York, and New York City), although not necessarily having the highest HPV vaccination coverage in the nation, have experienced annual increases in coverage that exceed the national average over a 4-year period. Activities contributing to this success, as reported by these immunization programs, include enhancing provider education, assessing vaccination coverage levels in health care provider offices and providing feedback to the practices, conducting media campaigns, engaging community partners, and experiencing a “spillover” effect from middle school vaccination requirements for Tdap and MenACWY vaccines.

At the end of 2016, the recommended HPV vaccination schedule was changed from a 3-dose to a 2-dose series for immunocompetent adolescents initiating the series before their 15th birthday. Three doses are recommended for persons initiating the series at ages 15 through 26 years and for immunocompromised persons (*2*). The recommendation allows for 1 fewer dose and one fewer visit to a health care provider, which might encourage providers to promote, and parents to accept, vaccination at the recommended age of 11–12 years. Although it is too early to assess the direct impact of the revised recommendation on vaccination practices, when applied retrospectively, the HPV up-to-date coverage was 6.3 percentage points higher than the ≥3-dose HPV coverage.

Each year in the United States, an estimated 31,500 newly diagnosed cancers in men and women are attributable to HPV; approximately 90% of these could be prevented by receipt of the nine-valent HPV vaccine (https://www.cdc.gov/cancer/hpv/statistics/cases.htm). Although it is too early to observe the impact of HPV vaccination on HPV-associated cancers, impact on infection with HPV types targeted by the vaccine and other endpoints have been reported (*6*–*8*). Data from the 2007–2010 National Health and Nutrition Examination Surveys indicate that, compared with 2003–2006 (before HPV vaccine introduction), prevalence of HPV types targeted by the quadrivalent HPV vaccine^†††^ in cervicovaginal specimens had decreased 56% (from 11.5% to 5.0%) among females aged 14–19 years (*6*). By 2011–2014, prevalence had declined 71% (from 11.5% to 3.3%) among females aged 14–19 years and 61% (from 18.5% to 7.2%) among females aged 20–24 years (*7*). Evidence of vaccine impact among males also exists (*8*).

The findings in this report are subject to at least five limitations. First, the overall household response rate was 32.7% (55.5% for the landline and 29.5% for the cell phone samples), and only 53.9% of landline-completed and 47.4% of cell phone–completed interviews had adequate provider data. Second, bias in estimates might remain even after adjustment for household and provider nonresponse and phoneless households.^¶¶¶^ Weights have been adjusted for the increasing number of cell phone–only households over time. Nonresponse bias might change, which could affect comparisons of estimates between survey years. Third, estimates stratified by state/local area might be unreliable because of small sample sizes. Fourth, multiple statistical tests were conducted, and a small number might be significant because of chance alone. Finally, ≥2-dose MenACWY coverage likely underestimates the proportion of adolescents who receive ≥2 MenACWY doses. Adolescents might receive a booster dose of MenACWY after age 17 years (*1*); because NIS-Teen includes adolescents aged 13–17 years, receipt of MenACWY at age ≥18 years cannot be captured in coverage estimates.

Adolescent vaccination coverage can be increased, and the gap between HPV vaccination coverage and coverage with Tdap and ≥1-dose MenACWY can be closed with increased implementation of effective strategies. Providers should use every visit to review vaccination histories, provide strong clinical recommendations for HPV and other recommended vaccines, and implement systems to eliminate or minimize missed opportunities (e.g., standing orders, provider reminders, patient reminder or recall, and use of immunization information systems) (https://www.thecommunityguide.org/topic/vaccination). Resources for clinicians to facilitate effective communication with parents and adolescents regarding HPV and other recommended vaccines are available at https://www.cdc.gov/hpv/hcp/index.html. Provider-based performance measurement could also facilitate increased adolescent vaccination coverage, including the Assessment, Feedback, Incentives, and eXchange program implemented by state and local immunization programs with individual providers (https://www.cdc.gov/vaccines/programs/afix/index.html), and the 2018 updated Healthcare Effectiveness Data and Information Set composite measure for health plans, assessing receipt of Tdap, MenACWY, and HPV vaccines by age 13 years (http://www.ncqa.org/hedis-quality-measurement/hedis-measures/hedis-2018). Protection against vaccine-preventable diseases will be increased if clinicians consistently recommend and simultaneously administer Tdap, MenACWY, and HPV vaccines at age 11–12 years.

**Summary**

**What is already known about this topic?**

To protect against vaccine-preventable diseases, including human papillomavirus (HPV)–associated cancers, diphtheria, pertussis, tetanus, and meningococcal disease, routine immunization of adolescents aged 11–12 years is recommended by the Advisory Committee on Immunization Practices (ACIP). Since HPV vaccine introduction in 2006 for females and 2011 for males, coverage has increased gradually for females and more rapidly for males, although coverage has not reached the tetanus, diphtheria and acellular pertussis vaccine (Tdap) and quadrivalent meningococcal conjugate vaccine (MenACWY) coverage.

**What is added by this report?**

In December 2016, ACIP updated HPV vaccination recommendations to include a 2-dose schedule for immunocompetent adolescents initiating the vaccine series before their 15th birthday; 3 doses are recommended for persons who initiate the series at age 15–26 years and for immunocompromised persons. A new HPV up-to-date measure was added to the 2016 National Immunization Survey–Teen to account for the revised HPV vaccination schedule. HPV up-to-date estimates were 49.5% for females and 37.5% for males and 6.0–6.5 percentage points higher than ≥3-dose adolescent HPV coverage. HPV up-to-date vaccination coverage was 15 percentage points lower among adolescents living in nonmetropolitan statistical areas (MSAs) than among adolescents living in MSA central cities.

**What are the implications for public health care?**

Adolescent vaccination coverage continues to improve, but opportunity remains to increase HPV-associated cancer prevention. A better understanding of reasons for differences in HPV vaccination by MSA status might identify appropriate strategies to improve coverage. Protection against vaccine-preventable diseases will be increased if clinicians consistently recommend and simultaneously administer Tdap, MenACWY, and HPV vaccines at age 11–12 years.

## Footnotes

* Eligible participants were born during January 1998–February 2004. Tetanus, diphtheria, and acellular pertussis vaccine (Tdap) represents coverage with ≥1 Tdap dose at age ≥10 years. Meningococcal conjugate vaccine (MenACWY) represents coverage with the quadrivalent meningococcal conjugate vaccine or meningococcal-unknown type vaccine. Coverage with meningococcal type B vaccine is not included in 2016 National Immunization Survey–Teen (NIS-Teen) vaccination coverage estimates. Human papillomavirus (HPV) vaccination coverage includes receipt of any HPV vaccine and does not distinguish between nine-valent (9vHPV), quadrivalent (4vHPV), or bivalent (2vHPV) vaccines. Some adolescents might have received more than the 2 or 3 recommended HPV vaccine doses. Estimates for hepatitis B and measles, mumps, and rubella vaccines represent coverage based on the catch-up schedule for adolescents who are not up to date with these vaccinations. Except as noted, coverage estimates for ≥1 and ≥2 varicella vaccine doses were obtained among adolescents with no history of varicella disease. Influenza vaccination coverage data are not included in this report but are available online at https://www.cdc.gov/flu/fluvaxview/index.htm.

† Most of the vaccination data for adolescents surveyed in the 2016 NIS-Teen were collected before publication of the 2-dose HPV schedule. However, the HPV up-to-date status of these adolescents was assessed to estimate what proportion would not need further HPV vaccination doses under the updated schedule. Adolescents were considered to be up to date with HPV vaccination if they had received ≥3 doses, or if each of the following applied: 1) they had received 2 doses; 2) the first dose was received before their 15th birthday; and 3) the difference between dates of first and second doses was ≥5 months minus 4 days, the absolute minimum interval between the first and second doses (https://www.cdc.gov/vaccines/programs/iis/cdsi.html).

§ Metropolitan statistical area (MSA) status was determined based on household-reported county of residence, and was grouped into three categories: MSA central city, MSA non-central city, and non-MSA. MSA and central city were as defined by the U.S. Census Bureau (https://www.census.gov/geo/reference/gtc/gtc_cbsa.html). Non-MSA areas include urban populations not located within an MSA as well as completely rural areas.

¶ Local areas that received federal Section 317 immunization funds were sampled separately: Chicago, Illinois; New York, New York; Philadelphia County, Pennsylvania; Bexar County, Texas; and Houston, Texas. Two local areas were oversampled: El Paso County, Texas and Dallas County, Texas. Three territories were sampled separately in 2016: Guam, Puerto Rico, and the U.S. Virgin Islands.

** All identified cellular-telephone households were eligible for interview. Sampling weights were adjusted for dual-frame (landline and cellular telephone), nonresponse, noncoverage, and overlapping samples of mixed telephone users. A description of NIS-Teen dual-frame survey methodology and its effect on reported vaccination estimates is available at https://www.cdc.gov/vaccines/imz-managers/coverage/nis/child/dual-frame-sampling.html.

†† For the telephone samples for the states and local areas, the overall Council of American Survey Research Organizations (CASRO) response rate was 32.7% (55.5% for the landline sample and 29.5% for the cellular telephone sample). For adolescents with completed interviews, 48.4% had adequate provider data (53.9% landline sample, 47.4% cell sample). Among completed interviews with adequate provider data, 23% (4,684) were from the landline sample and 77% (15,791) were from the cellular telephone sample. For territories, the overall CASRO response rates were 31.5% for Guam, 33.2% for Puerto Rico, and 44.4% for the U.S. Virgin Islands. The CASRO response rate is the product of three other rates: 1) the resolution rate (the proportion of telephone numbers that can be identified as either for business or residence); 2) the screening rate (the proportion of qualified households that complete the screening process); and 3) the cooperation rate (the proportion of contacted eligible households for which a completed interview is obtained).

§§ Adolescents from Guam (242 females and 293 males), Puerto Rico (197 females and 208 males), and the U.S. Virgin Islands (201 females and 227 males) were excluded from the national estimates.

¶¶ Adolescents were classified as below the federal poverty level if their total family income was less than the federal poverty level specified for the applicable family size and number of children aged <18 years. All others were classified as at or above the poverty level. Poverty status was unknown for 724 adolescents. https://www.census.gov/data/tables/time-series/demo/income-poverty/historical-poverty-thresholds.html.

*** Children aged ≤18 years who are Medicaid-eligible, uninsured, or American Indian/Alaska Native (as defined by the Indian Health Care Improvement Act) are eligible to receive vaccines from providers through the Vaccines for Children (VFC) program. Children categorized as “underinsured” (because their health plans do not include coverage for recommended vaccinations) are eligible to receive VFC vaccines if they are served by a rural health clinic or federally qualified health center or under an approved deputization agreement. https://www.cdc.gov/vaccines/programs/vfc/providers/eligibility.html.

††† Most HPV vaccine used in the United States before licensure of the nine-valent vaccine at the end of 2014 was quadrivalent HPV vaccine.

¶¶¶ In a sensitivity analysis of 2012 NIS-Teen data including adjustments for incomplete sample frame, nonresponse bias, and incomplete ascertainment of vaccination status, estimates of Tdap, ≥1-dose meningococcal conjugate, and ≥1-dose HPV vaccination coverage were estimated to be lower than actual values by 1–3 percentage points. https://www.cdc.gov/vaccines/imz-managers/nis/downloads/NIS-teen-puf15-dug.pdf.
